# The experiences of physiotherapists regarding self-management practices for patients with non-specific chronic low back pain, South Africa: A qualitative study

**DOI:** 10.4102/sajp.v82i1.2348

**Published:** 2026-09-22

**Authors:** Sergant G. Motha, Niri Naidoo, Karien Mostert

**Affiliations:** 1 Department of Physiotherapy, Faculty of Health Sciences, University of Pretoria, Pretoria, South Africa; 2 Department of Physiotherapy, Faculty of Health Sciences, University of Cape Town, Pretoria, South Africa

**Keywords:** non-specific chronic low back pain, self-management, physiotherapy experiences, biopsychosocial approach, qualitative research

## Abstract

**Background:**

Chronic non-specific low back pain (CNLBP) is one of the most common musculoskeletal conditions reported worldwide. Self-management interventions have been reported to be effective and affordable in managing CNLBP. However, few studies have been conducted in low- and middle-income countries, and their use by physiotherapists remains a global challenge.

**Objectives:**

This study aimed to gather physiotherapists’ input regarding self-management of CNLBP in the Limpopo province of South Africa.

**Method:**

A descriptive, exploratory qualitative study design was used. Physiotherapists working with CNLBP management were selected, and online interviews were conducted in English. Thematic analysis was used to analyse the data using ATLAS.ti software.

**Results:**

Twenty-one physiotherapists participated in interviews. Participants’ experiences were diverse. Six themes emerged: (1) Physiotherapists’ understanding of patients’ beliefs and practices, (2) Presentation of patients with CNLBP, (3) Elements of treatment, (4) Impact of social factors, (5) Accessibility to primary healthcare services, and (6) Interprofessional teamwork. These findings emphasise elements that must form part of a self-management programme.

**Conclusion:**

The biopsychosocial model of self-management, which includes a variety of exercises, educational programmes, socioeconomic factors, and interprofessional care, is esteemed as the most effective form of treatment and significantly improves access to primary healthcare.

**Clinical Implications:**

Healthcare professionals are advised to implement a biopsychosocial approach throughout the treatment process to enhance patient-centred care, taking into account patients’ beliefs and practices regarding CNLBP, its presentation, key elements of treatment, the role of social factors, access to services, and interprofessional teamwork.

## Background

Chronic non-specific low back pain (CNLBP) is the most common musculoskeletal condition globally, with a high prevalence and a leading cause of disability, especially in low- and middle-income countries (Traeger et al. 2019). The World Health Organization (WHO 2023) projected that the number of people with chronic low back pain (CLBP) will increase in the future, with the increase accelerating in low- and middle-income countries (Cieza et al. 2021; Traeger et al. 2019). Non-specific lower back pain is defined as a condition that is not attributable to a recognisable or known specific pathology (e.g. infection, tumour, fractures, structural deformity, inflammatory disorders, or radicular syndrome) (Wiechert, Wang & Chapman 2024). The WHO (2023) proposed non-drug, non-surgical approaches as first-line treatment for CLBP, including education on pain management and manual therapies. This type of rehabilitation is important for addressing the global burden of chronic musculoskeletal conditions, including CLBP (Cieza et al. 2021). A clinical guideline recommended the biopsychosocial approach and self-management interventions (SMIs) as the best management strategies for CNLBP (Almeida, Saragiotto & Maher 2018). Self-management interventions aim to equip patients with skills to actively participate and take responsibility in the management of their chronic condition to function optimally through at least knowledge acquisition and a combination of at least two of the following: stimulation of independent sign or symptom monitoring, medication management, enhancing problem-solving and decision-making skills for medical treatment management, and changing their physical activity or dietary or smoking behaviour (Jonkman et al. 2016) (Figure 1). However, the implementation of SMIs is challenging, particularly in low- and middle-income countries, due to the inaccessibility of healthcare centres (Hale et al. 2024). The SMI and biopsychosocial models are related to each other, as the biopsychosocial model is described as a patient-centred care framework that underpins the humanistic dimensions of its practical application, whereas SMI can be perceived as an empowering synthesis of an active biopsychosocial approach (Cormack et al. 2023). The biopsychosocial approach encompasses both biomedical and psychosocial elements, with biomedical elements generally aligning with the prevailing standard of care, including electrotherapy, myofascial release, and mobilisation (Van Dijk et al. 2023). Literature on the SMI approach for the management of chronic musculoskeletal conditions has been published; however, research on the self-management (SM) approach for CNLBP in low- and middle-income countries is lacking (Ahenkorah et al. 2019; Almeida et al. 2018; Malfliet et al. 2019; Mescouto et al. 2022; Oliveira et al. 2018). Limpopo province is part of South Africa, a low- and middle-income country, and has been classified as an impoverished region, comprising predominantly rural areas (Ngene, Khaliq & Moodley 2023). Physiotherapy plays an important role in improving the efficacy of interventions for chronic musculoskeletal pain, including CLBP, and has been extensively documented (Hutting et al. 2020). The implementation of psychologically informed physiotherapy is recommended to address psychosocial obstacles that may hinder successful outcomes in maintaining chronic musculoskeletal pain and SM (Diener 2021). Coronado et al. (2020) described psychologically informed physiotherapy as a rehabilitation approach that integrates psychological principles into physiotherapy treatment to address patient beliefs, emotions, and behaviours related to pain and movement. Additionally, reviews conducted in South Africa reported that clinicians are responsible for educating patients, communities, funders, policymakers, and other healthcare professionals on safe and effective management techniques within the SMI approach (Akhal et al. 2025; Hale et al. 2024). However, only one study on SMIs and physiotherapists conducted in South Africa was found; namely, a review of the use of physiotherapy to support SMIs (Diener 2021). The limited literature on SMIs is particularly important in countries such as South Africa, where disability rates are high and resources are limited. According to Jonkman et al. (2016), SMIs is a person-centred, collaborative approach used by healthcare providers to support individuals with health conditions in acquiring the necessary knowledge, skills, support, and confidence to play an active and leading role in managing their condition within the context of their daily lives. The SMIs should include both biomedical and psychosocial elements (Hutting 2024). Additionally, the systematic review by Akhal et al. (2025) outlined that education, community support, and mobile health support can be integrated into guided SMI for long-term conditions. The SMIs have been reported globally as the best option for the management of chronic musculoskeletal conditions; however, physiotherapists face challenges, such as limited resources and inaccessibility, in implementing SMI strategies in South Africa, (Diener 2021; Monk, Sole & Perry 2023). Hale et al. (2024) outlined the inaccessibility challenges as geographic disability, culture, ethnicity, language and communication, cognition, poverty, housing, income, employment insecurity, multimorbidity, and potentially living in low- and middle-income countries. These challenges result in a lack of implementation of the biopsychosocial, SMI, and psychologically informed physiotherapy approaches in South Africa. Additional studies are required to establish SMIs that may reduce the burden on physiotherapists, healthcare facility utilisation, and healthcare costs. Therefore, this study aimed to determine the perspectives of physiotherapists regarding SMI, identify gaps, and gather input to address these challenges. The results of this research are an integral component of an umbrella study to develop a self-management programme that may address the burden of CLBP by reducing the number of follow-up appointments for patients seen by physiotherapists in low- and middle-income countries, including South Africa.

**FIGURE 1:**
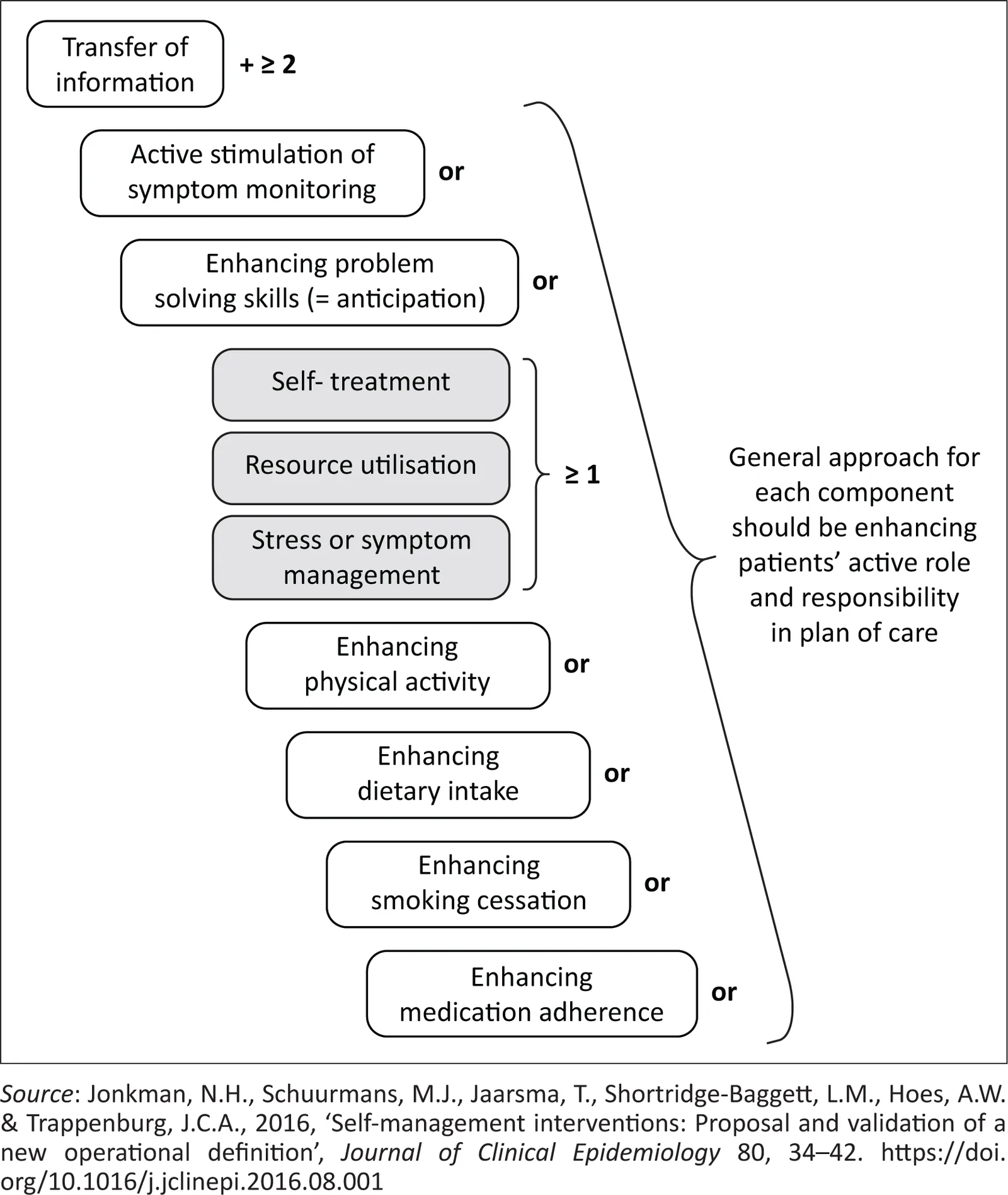
Self-management intervention.

## Research methods and design

A descriptive, exploratory qualitative study design was employed to gain a deeper understanding of the phenomenon of SMIs by examining the experiences of physiotherapists working with CNLBP (Creswell & Creswell 2018). The setting consisted of public hospitals in the Limpopo province with 140 physiotherapists distributed as seven assistant directors, nine chief physiotherapists, and 124 physiotherapists. Physiotherapy services were provided in outpatient, inpatient, and primary healthcare facilities, covering conditions across orthopaedics, neurology, surgery, and respiratory care. All participants aged 18 years and above were included regardless of their sex, duration of work experience, or postgraduate qualifications. Community-service physiotherapists and those who did not work with patients with back pain were excluded. Purposive sampling was used to recruit 36 participants, 26 of whom met the eligibility criteria. The following steps were used to recruit participants: (1) an invitation with study details was sent to the provincial physiotherapy coordinator to share with physiotherapists in Limpopo province public hospitals; (2) the participants contacted the researcher via email or WhatsApp to confirm their verbal consent to use their contact details; (3) after obtaining permission from the participants, study information with an informed consent brochure was sent via the Survey-Monkey platform (Maisaarah et al. 2018) and interview dates and times were scheduled via Doodle software (Labuda et al. 2017); (4) once all demographic information was obtained, a Zoom meeting link was sent to the participants. A reminder was sent via email 2 days before the interviews.

### Data collection

Semi-structured interviews were conducted with open-ended questions developed under the guidance of a contextual (Figure 2) and biopsychosocial model by selecting the relevant concepts referring to the study, that is, biological, psychological, social, physical, and treatment (Figure 3) (Bolton 2023; Goh et al. 2015). The interview guide consisted of Parts A and B (Online Appendix 1). Part A consisted of questions on demographic information (age, sex, work experience, and qualifications), and Part B included questions about physiotherapists’ experiences of SMI, such as patients’ beliefs about the causes of CLBP and strategies of SM. The first draft of the interview guide was sent to two academic physiotherapists who were experts in musculoskeletal physiotherapy for input, and the authors discussed finalising the guide with minor changes. An online interview pre-test was conducted with three physiotherapists. The interview guide with informed consent was reviewed to ensure the questions were clear and understandable, as well as to determine the interview duration (Buschle, Reiter & Bethmann 2022). The participants suggested a few amendments to the demographic section, information leaflets, and informed consent forms. The interviews were conducted in English by the first author with assistance from a trained research assistant. Prior to the interviews, the role of the research assistant was clarified thoroughly. All interviews were recorded with an average duration of approximately 44 min each. Otter and Zoom software were used for recording and transcription (Bokhove & Downey 2018). In addition, the first author took detailed notes during the interviews. Data collection was terminated when saturation was reached (no new information emerged from the interviews) (Hennink, Kaiser & Marconi 2017).

**FIGURE 2:**
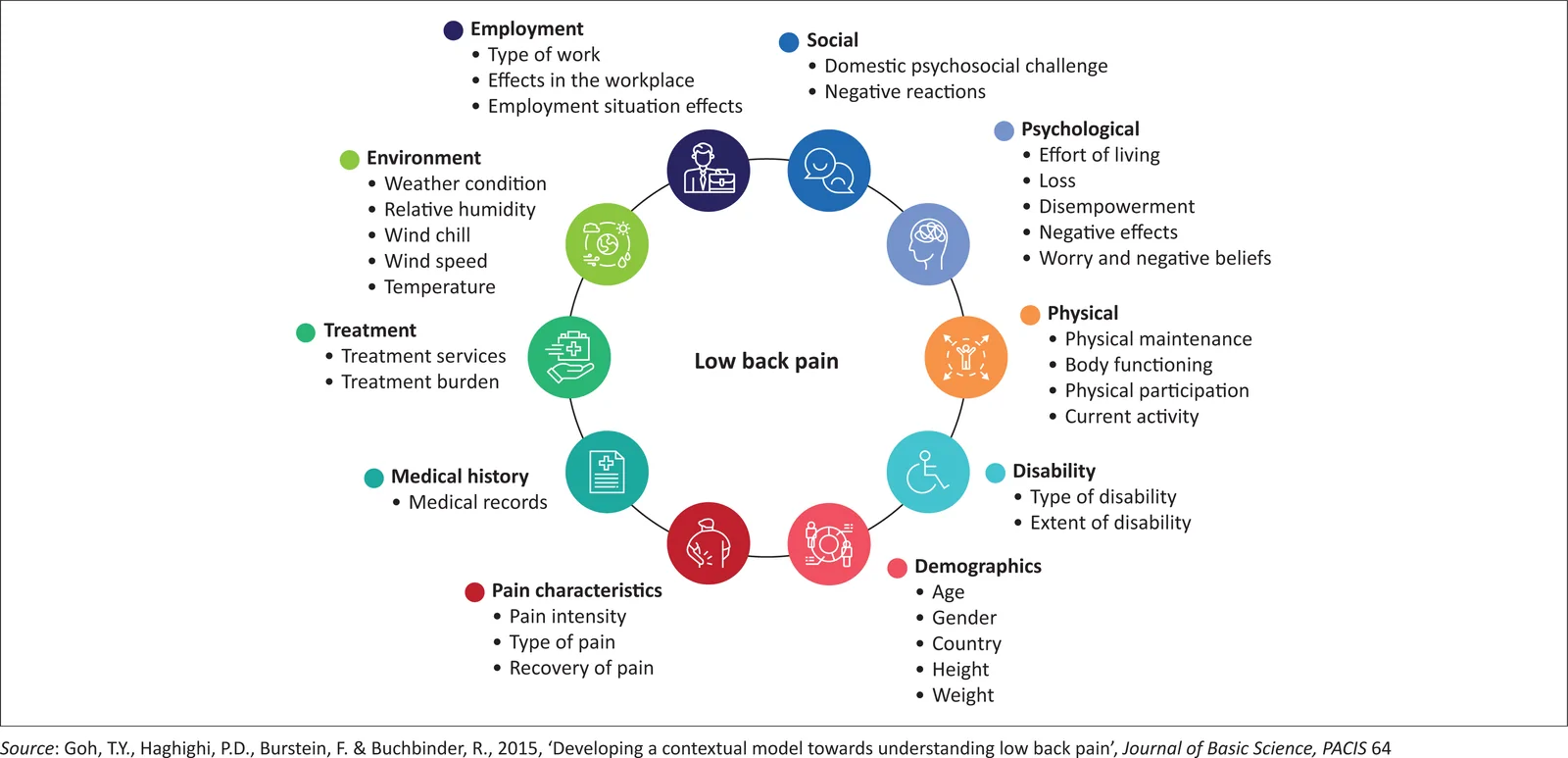
Contextual model for chronic low back pain.

**FIGURE 3:**
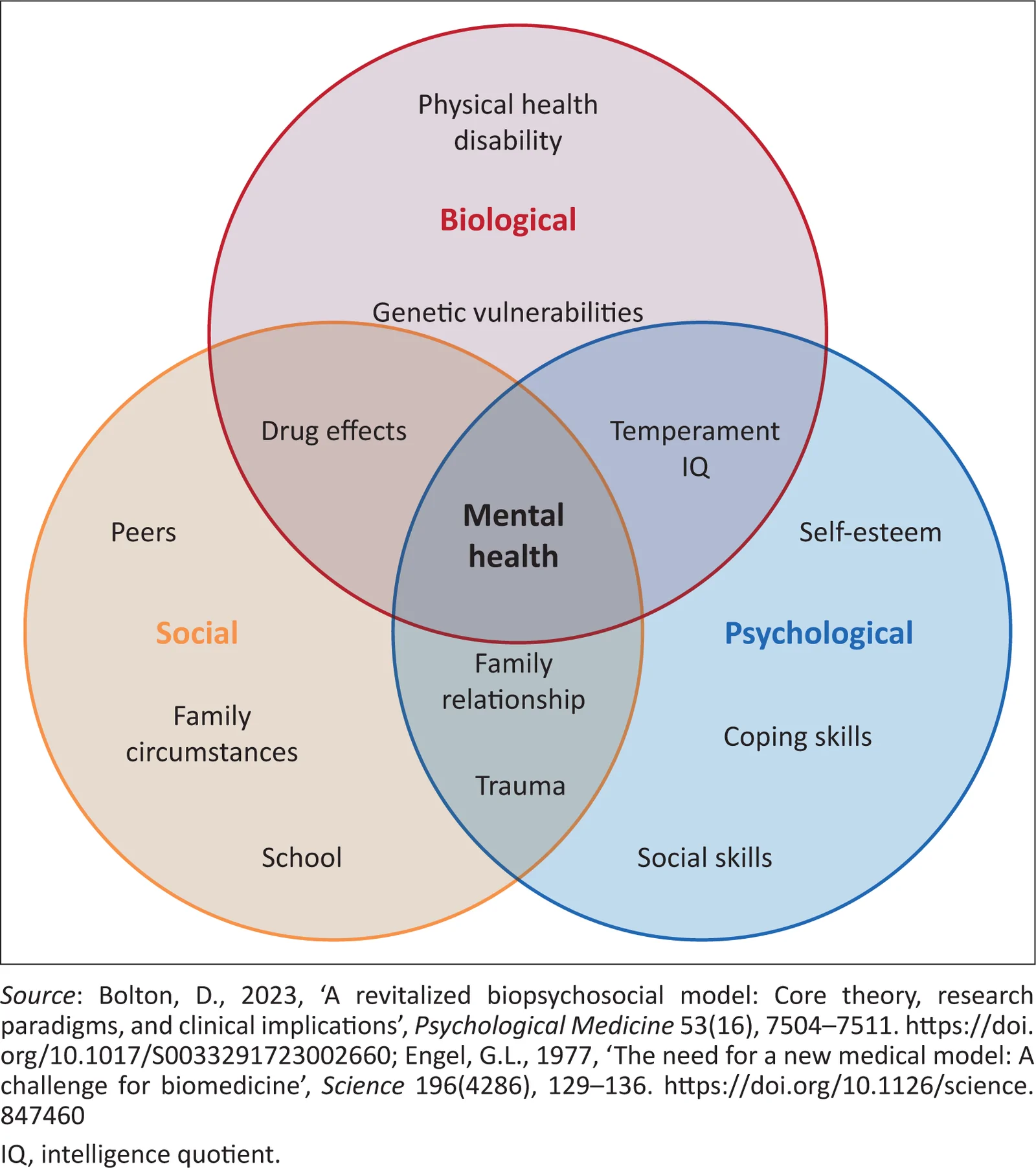
Biopsychosocial model for chronic low back pain.

### Data analysis

Descriptive statistics were used to summarise the demographic data in Stata version 17 (StataCorp LLC, College Station, TX, United States [US]). The interviews were transcribed verbatim and imported into ATLAS.ti version 2024 (ATLAS.ti Scientific Software Development GmbH, Berlin, Germany). Thematic analysis was used to identify, analyse, and report themes (Saldaña 2016). This process was used to understand the participants’ experiences by transforming the text into meaningful units, codes, categories, and themes (Naeem et al. 2023; Saldaña 2016). Our study used the following five steps to present the detailed actions: (1) The authors read the transcripts several times to understand the participants’ main ideas. After ATLAS.ti software artificial intelligence (Al) coding, a concept search was performed to identify keywords; (2) Three transcripts were coded inductively by the first author, followed by discussion with the other authors on the codes and categories. A coding framework was developed and used to code the remaining transcripts. Additional codes were created as necessary. A qualitative data analysis expert also independently analysed all transcripts; (3) Categorisation of codes and an expert independent code report were used to develop a draft of the code groups and sub-themes. This was followed by a discussion with the other authors, and the group codes and sub-themes were finalised; (4) Categorisation of the code groups was used to develop a draft of the themes, followed by discussion; and (5) The whole team extracted and discussed the main themes and illustrative quotes related to each theme. Measures were taken to ensure the trustworthiness of the data, focusing on key criteria such as credibility, dependability, confirmability, and transferability (Ahmed 2024). The authors conducted debriefing sessions throughout the research process to deliberate on the findings and data analysis. The code recording was completed, and the final categories were compared with the initial analysis. Regarding confirmability, all interviews were audio-recorded, field notes were kept, and reflexive analyses of the interviews were conducted. The digital software package ATLAS.ti was used for the data analysis to ensure transferability and confirmability. Credibility was established by engaging a qualitative data analysis expert and utilising two independently coded reports, which were then integrated.

### Ethical considerations

Ethical clearance was granted by the Research Ethics Committee, University of Pretoria, which complies with the Helsinki guidelines (Ethics reference no. 514/2021), and informed consent was obtained. Permission to use the study sites in the Department of Health, Limpopo, was obtained from the relevant authorities. This study was registered in the South African National Health Research Database (LP202110014).

## Results

### Part A: Demographic profile of the participants

Of the 26 recruited participants, 21 were interviewed (two participants resigned, two had network connection challenges, and one had time constraints and was therefore unavailable for the interviews) (Figure 4). Participant demographics indicated that five participants held postgraduate degrees or certificates in orthopaedic manual therapy, and one held a master’s degree in pain management. The youngest participant was 25 years old and the oldest 49 years old. Their years of work experience ranged from 6 years to 20 years. The participants were predominantly female (Table 1).

**FIGURE 4:**
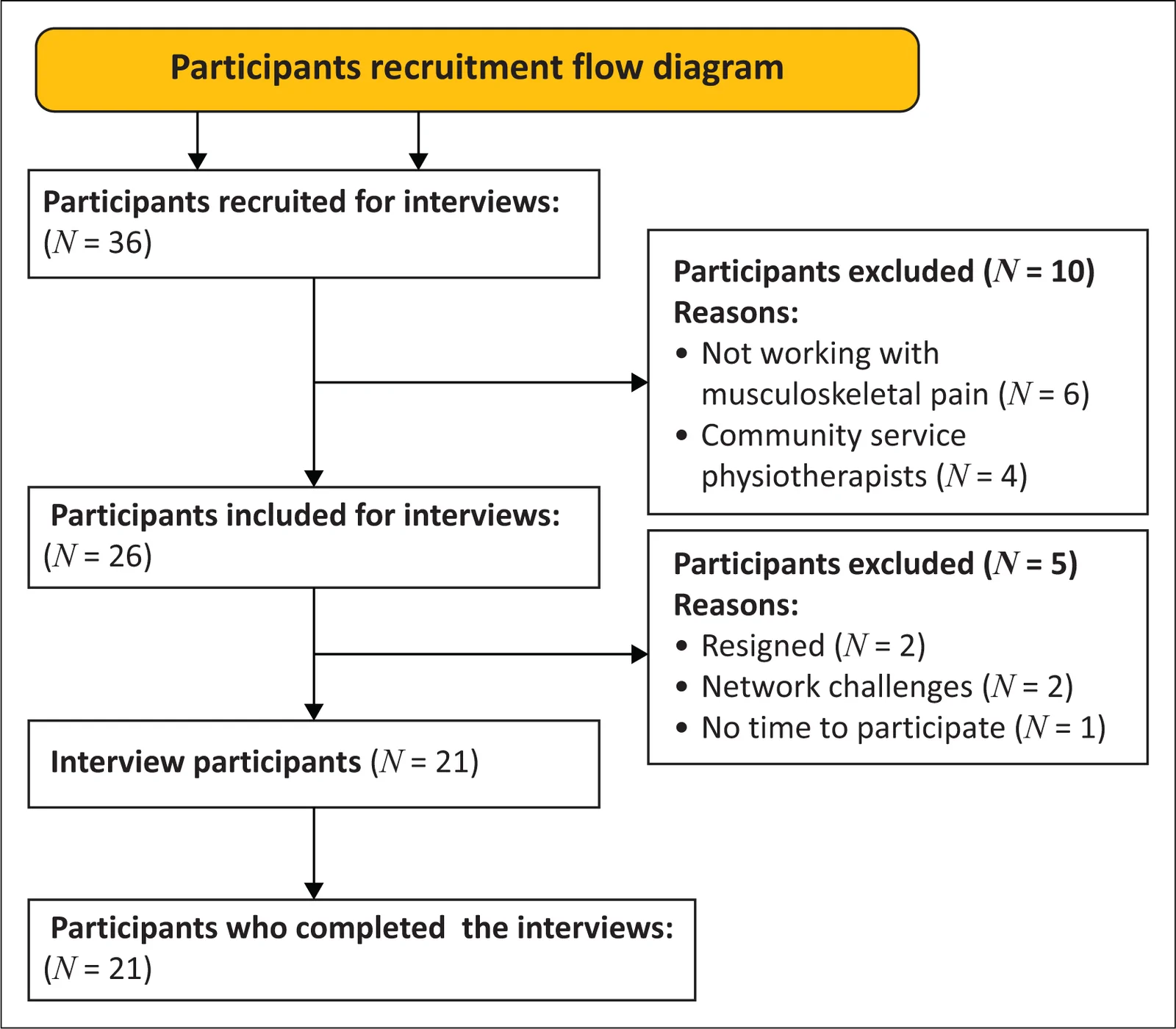
Participants’ recruitment flow diagram.

**TABLE 1:** Number of participants (*n* = 21).

Variables	Overall	
	Count (*n*)	%
**Sex**		
Male	3	14
Female	18	86
**Work setting**		
Rural	15	71
Urban	2	19
Both rural and urban	4	10

Note: Age (*M* ± s.d.) = 36.19 ± 4.56. Years of working experience (*M* ± s.d.) = 12.52 ± 4.68.

s.d., standard deviation; *M*, mean.

This interview aimed to gather perspectives from physiotherapists to gain a deeper understanding of their experiences with adults afflicted by CLBP. The goal is to assist in the formulation of an SMI programme that would help to reduce the rate of physiotherapy consultations in public hospitals.

### Part B: Experiences of physiotherapists on self-management

The themes that emerged from the analysis of the physiotherapists’ experiences with SM of CLBP were patient beliefs and practices, presentation of patients for physiotherapy, elements of treatment approaches, the impact of social factors, patient accessibility to primary healthcare (PHC) services, and interprofessional teamwork (see the coding summary, Table 2). Data collection continued until thematic saturation was reached, defined as the point at which no new codes or themes emerged from the interviews. Following analysis of 18 interviews, no new information regarding participant experiences was identified; therefore, data collection ceased at 21 participants, as further interviews were deemed redundant.

**TABLE 2:** Summary of themes and subthemes of physiotherapy interviews.

Themes	Sub-themes	Quotations	References
Theme 1: Physiotherapists’ understanding of patient beliefs and practices	Patient’s beliefs about treatment	‘In my experience most of the back pain patients try to manage themselves before they consult for assistance. They consult physiotherapy very late when they have tried everything, and their hopes remain to be physiotherapy.’	Participant 5, male, 33 years old, 11 years of physiotherapy experience, working in an urban area.
	Patient beliefs about causes	‘Sometimes they just don’t know what is happening with them but, they explained that they’re experiencing a very bad pain, and they unfortunately cannot link it to any incident or any trauma. ‘	Participant 3, female, 41 years old, 20 years of physiotherapy experience, rural practice.
		‘My experience in [working with] patients with chronic pain, [that it is] work-related, especially with those who are doing manual labour, for example, in the industry, or construction.’	Participants 12, female, 39 years old, 14 years of physiotherapy experience, working in an urban area
		‘They believe in traditional healers as they believe their back is caused by someone through witchcraft; then they feel that the hospital might not help.’	Participants 2, female, 40 years old, 19 years of physiotherapy experience, working in a rural area
Theme 2: Presentation of patients for physiotherapy	Physiotherapists’ experience working with CLBP	‘The institution that I’m [*the physiotherapist*] working at is more in the rural areas. I used to work in the Eastern Cape in a very rural hospital, and the kinds of cases [*the low back pain cases*] we saw were mostly malingering and that creates an environment where you are not sure if is really malingering or no case.’	Participant 14, female, 32 years old, 8 years of physiotherapy experience, working in a rural area
		‘With my experience, patients who are referred with back pain really vary in terms of how they present, and a lot of patients tend to become dependent over time. ‘	Participant 1, female, 31 years old, 6 years of working experience, working in a rural area
	Importance of the subjective examination	‘The subjective examination will help the therapist to identify if there are psychological factors and it is also important to give a patient an opportunity to present his or her problem and the goals.’	Participant 10, female, 37 years old, 13 years of working experience, working in a rural area
		‘When you do subjective examinations, trying to get the history of the condition, we notice that when we touch on the psychosocial aspects, most of them present with a whole lot of yellow flags.’	Participant 12, female, 39 years old, 14 years of working experience, working in a rural area
Theme 3: Elements of treatment approaches	Exercises	‘When you prescribe the exercise programme a demonstration must be done and [and you must] ask them if they understand.’	Participant 6, female, 30 years old, 6 years of working experience, working in an urban area
		‘The exercise programme should be easy and fun for different age groups in that way we can change the patient mindset about the exercise.’	Participant 18, female, 35 years old, 8 years of working experience, working in a rural area
	Education	‘I think education is the most important SM skill because the patients need to be taught their condition so that they’re able to handle themselves at home.’	Participant 8, female, 40 years old, 17 years of working experience, working in a rural area
		‘Maintaining and understanding the importance of posture and knowing how to correct your posture in general positions - sitting, standing, and whenever you’re performing the task. More emphasis must be placed on prevention, because it is better to prevent back pain. It is, therefore, important to teach the patients about correct lifting techniques and teach the children how they can carry their schoolbags.’	Participant 4, female, 41 years old, 20 years of working experience, working in both rural and urban
	Monitoring self-management	‘To make sure that the patient is adherent to the treatment is education, feedback, follow-up appointment, reminder [*use alarms, use pamphlets and videos*].’	Participant 9, female, 45 years old, 23 years of working experience, working in both rural and urban
		‘The telephone calls can be made to monitor progress or to find out if there are challenges, this would be possible if the resources allow it.’	Participant 13, female, 35 years old, 12 years working experience, working in a rural area
		‘Self-management is very important because we do not want the patient to rely 100% on physiotherapists; they need to take responsibility for their condition so that they can manage themselves at home, for example, to give them [*the patients*] handouts for home exercise as self-management.’	Participants 17, male, 31 years old, 10 years of working experience, working in a rural area
		‘Self-care will be much better and more affordable for them.’	Participant 21, male, 38 years old, 14 years of working experience, working in a rural area
	Patient discharge reports	‘We can use specific functional activities that we will rate each time they come. For example, they will tell you how far they are, how much they’ve improved with that activity, and whether they’re still struggling or improving at home. ‘	Participant 7, female, 41 years old, 18 years of working experience, working in both rural and urban area
		‘They reported that they were better for maybe five days after treatment.’	Participant 17, male, 31 years old, 10 years of working experience, working in a rural area
Theme 4: Impact of social factors	Identification of yellow flags	‘We have people who need the frequent appointments, for example, weekly appointments but unfortunately, they must wait for their payday to come again. So, it really affects the treatments’ progress because now they must wait for a month again to come for their next session.’	Participant 16, female, 33 years old, 10 years of working experience, working in a rural area
		‘In terms of socioeconomics, you will find that the lack of income, especially for the breadwinners, ends up affecting them, for example, you find that they end up just complaining of even the slightest pain that they’re having in their bodies, and they end up looking for disability compensation grant as they feel that they cannot work due to pain.’	Participant 18, female, 35 years old, 8 years of working experience, working in a rural area
	Patient support systems	‘The involvement of family members in the programme is important because all skills that are taught and preventative measures can be shared within the family, and they can also motivate, support, and remind each other about the programme.’	Participant 5, male, 33 years old, 11 years of working experience, working in a rural area
		‘Support groups among the patients can help the patients to educate and support each other within a nearby facility, clinic, home visit, or hospital. I think support groups can help to motivate one another also as a reminder.’	Participant 20, female, 36 years old, 10 years of working experience, working in a rural area
Theme 5: Patient accessibility to primary healthcare (PHC) services	Treatment accessibility	‘Some patients are familiar with technology, so the video exercises can be shared, and there are exercise apps that can be used. This can be used by patients among each other or with the physiotherapist with the use of WhatsApp.’	Participant 6, female, 30 years old, 6 years of working experience, working in a rural area
		‘The socioeconomic problems are affecting most people’s access to things like smart cell phones, Wi-Fi, so the government needs to intervene to ensure accessibility. The government needs to look into it, because that will assist in a whole lot of issues besides this topic, for example, collecting the medicine, a cell phone can be the easiest way to just know when you can collect.’	Participant 11, female, 37 years old, 13 years of working experience, working in a rural area
	Outreach services	Outreach services can also help us to reach a whole lot of people by making sure that we follow up on some of the things that we would have done within the facility itself.’	Participant 19, female, 32 years old, 9 years of working experience, working in a rural area
		‘The home visits are also done to ensure that everything is working around their homes, and they need to change around to make sure that it’s it works for them.’	Participant 15, female, 29 years old, 6 years of working experience, working in a rural area
Theme 6: Interprofessional teamwork	Interprofessional team needed to assist patient to cope	‘The biopsychosocial approach is a multi-modal treatment [*approach*] for pain, including both biomedical and psychosocial [*domains*]. Therefore, if those components have been identified through the assessments, they need to be addressed, which means an interdisciplinary pain management team should [*work together*], for example, doctors, dietitians, psychologists and occupational therapists.’	Participant 11, female, 37 years old, 13 years of working experience, working in a rural area

CLBP, chronic low back pain; SM, self-management.

#### Theme 1: Physiotherapists’ understanding of patient beliefs and practices

**Patients’ beliefs about treatment:** The physiotherapists reported that patients have limited knowledge of the role of physiotherapy in managing CLBP. This resulted in patients consulting physiotherapists very late, and they ultimately became dependent on physiotherapy. Some participants reported late patient consultation because of their cultural practices (for example, consulting traditional healers) and beliefs that resulted in longer times to recover:

‘In my experience, most of the back pain patients try to manage themselves before they consult for assistance. They consult physiotherapy very late when they have tried everything, and their hope remains in physiotherapy.’ (Participant 5, male, 33 years old, 11 years of physiotherapy experience, working in an urban area)

**Patient beliefs about the causes of chronic low back pain:** Physiotherapists reported that patients believe that there are various causes of their CLBP, but some were not easily identifiable, traceable, or clear, making it difficult for them to understand their condition. Some participants reported that most patients were unaware of the cause of their back pain:

‘Sometimes they just don’t know what is happening with them, but they explain that they’re experiencing very bad pain, and they unfortunately cannot link it to any incident or any trauma.’ (Participant 3, female, 41 years old, 20 years of physiotherapy experience, rural practice)

Most physiotherapists reported that, in their experience, they encountered most of the CLBP cases resulting from work or activities of daily living:

‘My experience in [*working with*] patients with chronic pain, is [*that it is*] work-related, especially with those who are doing manual labour, for example, in the industry, or construction.’ (Participants 12, female, 39 years old, 14 years of physiotherapy experience, working in an urban area)

Some physiotherapists reported that some patients believed that witchcraft caused their back pain:

‘They believe in traditional healers as they believe their back is caused by someone through witchcraft; then they feel that the hospital might not help.’ (Participants 2, female, 40 years old, 19 years of physiotherapy experience, working in a rural area)

#### Theme 2: Presentation of patients for physiotherapy

**Physiotherapists’ experiences of chronic low back pain patient presentation:** Physiotherapists have different experiences with patients in rural versus urban settings. For example, one participant reported working in a rural area, where CLBP was among the most challenging conditions to manage:

‘The institution that I am working at is in the rural areas. I used to work in the Eastern Cape in a very rural hospital, and the kinds of cases we saw were mostly malingering, and that creates an environment where you are not sure if it is really malingering or not.’ (Participant 14, female, 32 years old, 8 years of physiotherapy experience, working in a rural area)

Furthermore, the participants reported that, based on their experience working in rural areas, patients tend to rely heavily on physiotherapy:

‘With my experience, patients who are referred with back pain, really vary in terms of how they present, and a lot of patients tend to become dependent with time.’ (Participant 1, female, 31 years old, 6 years of working experience, working in a rural area)

**Importance of the subjective examination:** The subjective patient examination plays a crucial role in patient management, particularly for chronic pain, as it provides guidance for further examination and treatment of the psychological aspects of pain:

‘The subjective examination will help the therapist to identify if there are psychological factors and it is also important to give a patient an opportunity to present his or her problem and the goals.’ (Participant 10, female, 37 years old, 13 years of working experience, working in a rural area)

‘When you do subjective examinations trying to get the history of the condition, we notice that when we touch on the psychosocial aspects, most of them present with a whole lot of yellow flags.’ (Participant 12, female, 39 years old, 14 years of working experience, working in a rural area)

#### Theme 3: Elements of treatment approaches

Physiotherapists reported that various treatment approaches have been employed to manage CLBP, including exercise, education, SM monitoring, and patient discharge reporting.

**Exercises:** The physiotherapists divulged that the prescribed exercise programme should be easy to use in order to manage CLBP at home. Most participants reported that when performing individual exercises, the following should be considered:

‘When you prescribe the exercise programme a demonstration must be done and ask them if they understand.’ (Participant 6, female, 30 years old, 6 years of working experience, working in an urban area)

‘The exercise programme should be easy and fun for different age groups in that way we can change the patient mind-set about the exercise.’ (Participant 18, female, 35 years old, 8 years of working experience, working in a rural area)

**Education:** Physiotherapists communicated that education plays a major role in the management of CLBP, including education about the condition and the importance of treatment compliance:

‘I think education is the most important SM skill because the patients need to be taught their condition so that they’re able to handle themselves at home.’ (Participant 8, female, 40 years old, 17 years of working experience, working in a rural area)

The physiotherapists outlined that posture awareness can also benefit patients with CLBP:

‘Maintaining and understanding the importance of posture and knowing how to correct your posture in general positions – sitting, standing, and whenever you’re performing the task. More emphasis must be placed on prevention, because it is better to prevent back pain. It is, therefore, important to teach the patients about correct lifting techniques and teach the children how they can carry their schoolbags.’ (Participant 4, female, 41 years old, 20 years of working experience, working in both rural and urban)

**Monitoring self-management:** The participants relayed that various methods, such as telephone calls, face-to-face and follow-up appointments, can be used to ensure adherence to a home treatment programme. The participants reported receiving follow-up feedback, making telephone calls, and using videos to ensure adherence to the home exercise programme:

‘To make sure that the patient is adherent to the treatment is education, feedback, follow-up appointment, reminder [*use alarms, use pamphlets and videos*].’ (Participant 9, female, 45 years old, 23 years of working experience, working in both rural and urban)

‘… telephone calls can be made to monitor progress or to find out if there are challenge; this would be possible if the resources allow it.’ (Participant 13, female, 35 years old, 12 years working experience, working in a rural area)

‘Self-management is very important because we do not want the patient to rely 100% on physiotherapists; they need to take responsibility for their condition so that they can manage themselves at home, for example, to give them [*the patients*] handouts for home exercise as self-management.’ (Participants 17, male, 31 years old, 10 years of working experience, working in a rural area)

‘Self-care will be much better and affordable for them.’ (Participant 21, male, 38 years old, 14 years of working experience, working in a rural area)

**The patient discharge reporting:** The physiotherapists reported that a feedback report after the patient had been discharged would guide the treatment or the next step to be taken when reviewing treatment. The physiotherapists revealed that feedback reports after discharge helped improve the tasks or exercises given in the home programme:

‘We can use specific functional activities that we will rate each time they come. For example, they will tell you how far they are, how much they’ve improved with that activity, and whether they’re still struggling or improving at home.’ (Participant 7, female, 41 years old, 18 years of working experience, working in both rural and urban area)

#### Theme 4: Impact of social factors

**Identification of yellow flags:** Social factors, such as financial status, can influence treatment effectiveness in patients with CLBP. The physiotherapists also reported that the main problem that patients experienced was often related to social challenges, as patients did not have the means, amongst others, to attend physiotherapy as required:

‘We have people who need the frequent appointments, for example, weekly appointments but unfortunately, they must wait for their payday to come again. So, it really affects the treatments’ progress because now they must wait for a month again to come for their next session.’ (Participant 16, female, 33 years old, 10 years of working experience, working in a rural area)

Physiotherapists also reported that some patients were attending physiotherapy continuously without showing improvement, as they were primarily seeking financial compensation, specifically a disability grant:

‘In terms of socioeconomics, you will find that the lack of income especially for the breadwinners, end up affecting them, for example, you find that they end up just complaining of even the slightest pain that they’re having in their bodies, and they end up looking for disability compensation grant as they feel that they cannot work due to pain.’ (Participant 18, female, 35 years old, 8 years of working experience, working in a rural area)

**Patient support systems:** Physiotherapists outlined that social support from a family member, caregiver, or community can positively influence the outcome of treatment for patients with CLBP, and negatively when there is no support. The participants reported that the involvement of a caregiver and family contributed to patients’ improvement, as they provided support:

‘The involvement of family members in the programme is important because all skills that are taught and preventative measures can be shared within the family, and they can also motivate, support, and remind each other about the programme.’ (Participant 5, male, 33 years old, 11 years of working experience, working in a rural area)

Additionally, the physiotherapists reported that a support group also contributed to the patient’s improvement, as they motivated one another:

‘Support groups among the patients can help the patients to educate and support each other within a nearby facility, clinic, home visit, or hospital. I think support groups can help to motivate one another also as a reminder.’ (Participant 20, female, 36 years old, 10 years of working experience, working in a rural area)

#### Theme 5: Patient accessibility to primary healthcare services

**Treatment accessibility:** The physiotherapists communicated that digital interventions play a role in improving SM in patients with chronic pain. Therefore, accessibility to digital devices is important:

‘Some patients are familiar with technology, so video exercises can be shared, and there are exercise apps that can be used. This can be used by patients among each other or with the physiotherapist with the use of WhatsApp.’ (Participant 6, female, 30 years old, 6 years of working experience, working in a rural area)

However, some physiotherapists reported limited access to digital devices and emphasised the need to consider and support digital opportunities:

‘The socioeconomic problems are affecting most people’s access to things like smart cell phones, wireless networking technology [*Wi-Fi*], so the government needs to intervene to ensure accessibility. The government needs to look into it, because that will assist in a whole lot of issues besides this topic, for example, collecting the medicine. Cell phones can be the easiest way to just know when you can collect.’ (Participant 11, female, 37 years old, 13 years of working experience, working in a rural area)

**Outreach services:** Physiotherapists reported that out-of-hospital care, either at the clinic or during home visits, is beneficial to patients with CLBP, as it provides convenience for patients, as these services are rendered closer to or at patients’ homes:

‘Outreach services can also help us to reach a whole lot of people by making sure that we follow up on some of these things that we would have done within the facility itself.’ (Participant 19, female, 32 years old, 9 years of working experience, working in a rural area)

‘The home visits are also done to ensure that everything is working around their homes, and they need to change around to make sure that it works for them.’ (Participant 15, female, 29 years old, 6 years of working experience, working in a rural area)

#### Theme 6: Interprofessional teamwork

An interprofessional team is needed to assist with coping with chronic low back pain: The participants outlined the biopsychosocial approach as a method that uses both biomedical and psychosocial factors to cause or contribute to CLBP:

‘The biopsychosocial approach is a multi-modal treatment [*approach*] for pain, including both biomedical and psychosocial [*domains*]. Therefore, if those components have been identified through the assessments, they need to be addressed, which means an interdisciplinary pain management team should [*work together*], for example, doctors, dietitians, psychologists and occupational therapists.’(Participant 11, female, 37 years old, 13 years of working experience, working in a rural area)

## Discussion

This study obtained input from physiotherapists about self-management to explore their working experiences with adults with CLBP in Limpopo province, South Africa, to contribute to the development of an SM programme.

The main themes that emerged from the analysis were patients’ beliefs and practices, patients’ presentation of patients to physiotherapy, elements of treatment, the impact of social factors, patients’ access to PHC services, and patients’ coping mechanisms.

### Patient beliefs and practices

Most physiotherapists reported the causes of CNLBP as unknown. This statement is supported by the literature, in which CNLBP is defined as a musculoskeletal condition that is not attributable to a recognisable or known specific pathology and persists for more than 12 weeks (Wiechert et al. 2024). Work-related low back pain (LBP) is a leading cause of disability globally and creates a healthcare burden by increasing healthcare utilisation (Cieza et al. 2021). A review conducted by Maharjan et al. (2025) on work-related low back pain reported a high prevalence, ranging from 11% (during the 2 years after the first year of implementation) to 99.3% (at 5 years). This result supports the view that back pain can arise from work-related situations and scenarios. Regarding patients’ beliefs, participants emphasised facilitators and barriers, including cultural aspects and beliefs about cause and effect, as contributing factors to CLBP. Rodrigues-De-Souza et al. (2016) conducted a qualitative study on sociocultural factors and the experiences of patients with CLBP, stating that alternative treatments affect their experiences. These elements need to be incorporated into future SM programmes.

### Presentation of patients for physiotherapy

The physiotherapists working with CLBP should include a subjective examination during the treatment process, such as using patient-reported outcome measures, which would benefit the clinical reasoning process, increase the quality of assessment, obtain the patient’s voice, educate and motivate patients, and communicate with health professionals, to improve the consequent self-management (Östhols, Boström & Rasmussen-Barr 2019). Psychological aspects, such as yellow flags, have also been reported by participants as an important factor during subjective examinations in the management of CLBP. Furthermore, a qualitative systematic review outlined the benefits of individuality (‘Getting to know the patient’ and ‘Individualised treatment’), education, communication (‘Non-verbal communication’), goal setting, support (‘Empowerment’), social characteristics of a patient-centred physiotherapist, confident physiotherapist and patient-centred physiotherapy (Wijma et al. 2017).

### Elements of treatment approaches

The physiotherapists strongly supported self-management as an intervention. The self-management approach has been reported to be the best intervention for CLBP (Hutting 2024). A systematic review and meta-analysis of SMI strategies for chronic musculoskeletal pain conditions found that SMI reduced pain in the short and intermediate terms and disability in the long term (Cheng et al. 2025; Du et al. 2011, 2017). Exercises as part of self-management should be easy to perform, individualised and incorporated into daily activities. A randomised controlled trial conducted in New Zealand and Denmark showed that personalised exercise therapy and self-management support programmes were more effective than usual care alone (Skou et al. 2025). Personalised exercises were described as exercises that were prescribed based on individuals’ needs and progressed or regressed based on their progress. Education about the condition, including posture awareness and the importance of exercise in patients with CLBP, was outlined by most participating physiotherapists. A randomised controlled study comparing education alone and education plus therapeutic alliance (the collaborative bond between a therapist and a patient) showed that both interventions were effective (Miyamoto et al. 2021). Pain neuroscience education guided by a biopsychosocial approach has demonstrated greater efficacy in managing chronic musculoskeletal conditions, including CLBP, by reducing pain, improving patients’ knowledge of pain, improving function, reducing disability, reducing psychosocial factors, enhancing movement, and minimising healthcare utilisation (Cuenca-Martínez et al. 2023). Therefore, patients should be educated to adhere to SMIs.

The physiotherapists recommended various strategies to improve adherence to a self-management programme, including follow-up appointments, exercise handouts, feedback, telephone calls, and video exercises that can be used to promote compliance with self-management of CLBP. Peek et al. (2016) also found that activity monitoring and feedback, written exercise instructions, a behavioural exercise programme with booster sessions, and goal setting can aid patient adherence. Additionally, biopsychosocial factors, therapeutic skills and patient-physiotherapist collaboration are prerequisites that should be considered in physiotherapy practice to facilitate adherence in CLBP (Alt, Luomajoki & Luedtke 2023).

### Impact of social factors

Most participants reported working in a rural setting. Studies conducted in South Africa and America have outlined the challenges that have an impact on people living in rural areas compared with urban settings, which include health professional workforce shortages, delayed care (often travelling long distances for their care), and poor socioeconomic status (Ngene et al. 2023; Nielsen, D’Agostino & Gregory 2017). The caregiver’s role in supporting patient self-management is multidimensional, encompassing three processes of care (self-care maintenance, self-care monitoring and self-care management) and influenced by multiple factors in the management of chronic conditions (Schulman-Green et al. 2021). Some studies have outlined that supporting patients may involve managing multiple health domains, including physical and psychological symptom management, discussions of advance care planning, and providing social and financial support, as well as assistance with activities of daily living (Dineen-Griffin et al. 2019; Hale et al. 2024; Hutting et al. 2024). Additionally, patients’ health conditions should be closely monitored to adjust care, and patients should be advocated for and communicated with regarding health professionals and services on their behalf (Corry et al. 2015; Whitehead et al. 2018). This is consistent with participants’ views that family members or caregivers play a major role in the management of CLBP. There are barriers, such as socioeconomic factors and patients’ social support, that can negatively affect patient adherence to self-management (Boutevillain et al. 2017). Most participants reported the effect of financial constraints and patient social support on SMIs. Ngene et al. (2023) and Nielsen et al. (2017) showed that challenges faced by people residing in rural settings with high unemployment rates, limited health facilities and poor infrastructure can contribute to poor adherence to SM programmes. These challenges must be taken into account when developing treatment interventions in rural settings.

### Patient accessibility to primary healthcare services

Digital strategies of treatment have been reported by participants as a method that addresses socioeconomic challenges and improves adherence to SM programmes. A number of studies have reported digital strategies, such as telerehabilitation, video-based exercises, and cell phone apps, as the best intervention compared with face-to-face or traditional approaches in the SM of CLBP (Baroni et al. 2023; Chhabra, Sharma & Verma 2018; Özden et al. 2022). Furthermore, participants reported that home visits and outreach services could facilitate SM programmes. Despite socioeconomic challenges, the current literature supports the use of digital strategies. However, most of these studies were conducted in developed countries. To date, there is limited literature on the use of digital strategies in rural areas of low- and middle-income countries. Therefore, considering digital strategies in the implementation of SMI remains challenging. The participants reported that a biopsychosocial approach was the optimal option for managing CLBP. This statement is supported by several international treatment guidelines for CLBP, which endorse the biopsychosocial approach as the best option (Cormack et al. 2023; Corp et al. 2021; Oliveira et al. 2018). Cormack et al. (2023) reviewed the biopsychosocial model as a patient-centred care framework that underpins the humanistic dimensions of its practical application, which can be perceived as an empowering synthesis of an active biopsychosocial approach. According to Simpson et al. (2021), biopsychosocial consists of three components: biological (associated with the relationship between disease and body health), psychological (aspect of mental and emotional wellness that also relates to behaviour), and social (interpersonal factors, such as social interactions and community activities). This stance is consistent with participants emphasising the linkage between and the importance of psychological and social factors in the management of CLBP.

### Interprofessional teamwork

Participants also argued that an interprofessional team approach plays a significant role in implementing biopsychosocial treatment, for example, by referring to other professionals, such as psychologists and dietitians, as these professions are integral to the biopsychosocial model. The importance of an interprofessional team approach has also been outlined in recent WHO and European guidelines for managing CLBP in primary and community care settings (Alperovitch-Najenson et al. 2023). Therefore, for positive outcomes, the literature supports the use of the biopsychosocial model, incorporating a multi-professional team, to address the challenges experienced in CLBP.

## Conclusion

Physiotherapists supported the biopsychosocial approach and self-management programmes as the best treatment approach for the management of CNLBP. Self-management programmes should include various treatment types, including exercises, education, and addressing socioeconomic factors.

The biopsychosocial model of self-management, which encompasses a variety of exercises, educational programmes, socioeconomic factors, and interprofessional care, is widely regarded as the most effective approach to treatment and could significantly improve access to primary healthcare.

### Limitation

The following limitation is acknowledged: The results of this study cannot be generalised as the participants were from a specific setting. However, readers may evaluate whether their context is similar to that of this area in the Limpopo province.

### Clinical implications

To enhance patient-centred care, it is recommended that healthcare professionals working in rural environments consistently employ the biopsychosocial approach throughout the treatment process. In addition, they should identify and address the challenges encountered during implementation.

### Recommendations

There is a need for more studies on physiotherapy-led self-management and its impact on CLBP. Healthcare providers should be encouraged to follow the biopsychosocial model when implementing SM for CLBP. We also recommend further research on the implementation of a digital approach to SMIs and the development of policies to address the socioeconomic challenges in low- and middle-income countries. The perspectives of patients should also be determined to enhance the self-management strategies for patients with CLBP.
